# The Elaborate Postural Display of Courting *Drosophila persimilis* Flies Produces Substrate-Borne Vibratory Signals

**DOI:** 10.1007/s10905-016-9579-8

**Published:** 2016-09-02

**Authors:** Mónica Vega Hernández, Caroline Cecile Gabrielle Fabre

**Affiliations:** Department of Zoology, University of Cambridge, Downing Street, Cambridge, CB2 3EJ UK

**Keywords:** Biotremology, *drosophila*, *persimilis*, *pseudoobscura*, courtship, behavior, abdomen, tremulation, substrate-borne vibrations, female stationary, quivering, feeding, copulation

## Abstract

Sexual selection has led to the evolution of extraordinary and elaborate male courtship behaviors across taxa, including mammals and birds, as well as some species of flies. *Drosophila persimilis* flies perform complex courtship behaviors found in most *Drosophila* species, which consist of visual, air-borne, gustatory and olfactory cues. In addition, *Drosophila persimilis* courting males also perform an elaborate postural display that is not found in most other *Drosophila* species. This postural display includes an upwards contortion of their abdomen, specialized movements of the head and forelegs, raising both wings into a “wing-posture” and, most remarkably, the males proffer the female a regurgitated droplet. Here, we use high-resolution imaging, laser vibrometry and air-borne acoustic recordings to analyse this postural display to ask which signals may promote copulation. Surprisingly, we find that no air-borne signals are generated during the display. We show, however, that the abdomen tremulates to generate substrate-borne vibratory signals, which correlate with the female’s immobility before she feeds onto the droplet and accepts copulation.

**Electronic supplementary material:**

The online version of this article (doi:10.1007/s10905-016-9579-8) contains supplementary material, which is available to authorized users.

## Introduction

A variety of animals perform courtship displays to attract a mate. The complexity of these displays is driven by sexual selection, which in some species has led to extraordinary patterns of male courtship (Andersson 1994). For example, during courtship, the male bird of paradise presents an iridescent chest and snaps its tail feathers rhythmically while hopping on its legs (Cooper and Forshaw 1977). In jumping spiders and wolf spiders, courting males raise their abdomen and/or legs to attract the female’s attention (Waldock 1993; Hebets and Uetz 2000; Girard and Endler 2014; Girard et al. 2015). These behaviors provide visual signals to the females (Cooper and Forshaw 1977; Amundsen et al. 1997; Bennett et al. 1997; Pearn et al. 2001; Hausmann et al. 2003; Li et al. 2008; Lim et al. 2008; Uhl and Elias 2011), but, at the same time, they may also generate cues such as air-borne or substrate-borne signals (Edwards 1981; Gwynne and Dadour 1985; Maddison and Stratton 1988a, 1988b; Read and Weary 1992; Elias et al. 2003; Sivalinghem et al. 2010; Girard et al. 2011; Uhl and Elias 2011). The females use all of these pieces of information to assess males and to decide whether to accept copulation. It was long thought that the jumping spider females assessed the courting display of the males mainly visually (Foelix 1996). In some species, courting males were shown to also produce air-borne sounds [see for example (Edwards 1981; Gwynne and Dadour 1985)]. Later, the abdominal movements of the males were shown to produce substrate-borne vibrations; the transfer of these vibrations through the ground is essential for the copulation success of the males (Elias et al. 2003, 2005, 2006, 2010; Sivalinghem et al. 2010). Substrate-borne vibrational communication is widely used by animals, in particular by invertebrates, and has recently received increased attention in the literature (Virant-Doberlet and Cokl 2004; Cocroft and Rodríguez 2005; Polajnar et al. 2014; Hill and Wessel 2016; Polajnar et al. 2016; Rebar and Rodríguez 2016). Identifying and monitoring these vibrations that are imperceptible to humans requires sophisticated technologies, such as laser vibrometry (Elias et al. 2003; Cocroft and Rodríguez 2005; Girard et al. 2011).

*Drosophila persimilis* flies are a typical wild inhabitant of the western United States, where they breed on the infected sap of trees. Although their behaviour and ecology are not completely understood, *D. persimilis* flies are usually found together with the almost morphologically indistinguishable species *Drosophila pseudoobscura* (Carson 1951). Like other *Drosophila* species the *D. persimilis* males produce “standard” courtship behaviors, including the wing fluttering that produces an air-borne sound involved in species recognition (Spieth 1952; Brown 1965; Greenspan and Ferveur 2000). Remarkably, *Drosophila persimilis* is one of the few *Drosophila* species, in which males also exhibit an elaborate postural display in addition to the standard courtship steps (Spieth 1952). Some parts of this postural display were noted previously, including the upward movement of the wings (“wing-posture”), the rowing of one leg, and the dramatic rise of the abdomen and the forelegs (Brown 1965). Nevertheless, several aspects of the description and signals produced by this postural display, as well as the behavior of the female during the display, remain unknown. It has been suggested that this postural display produces mainly visual cues, and may also produce air-borne cues (Brown 1964, 1965). Here, we revisit *D. persimilis* courtship mating behavior and we describe the postural display using modern imaging and sophisticated recording techniques to ask which non-visual signals might promote copulation.

## Materials and Methods

### Flies

*Drosophila persimilis (*UC San Diego Drosophila stock center, stock number 14,011–0111.00, collected from Cold Creek, California*), D. pseudoobscura* (UC San Diego Drosophila stock center, stock number 14,011–0121.00, collected from Tucson, Arizona) and *D. melanogaster* Canton-S flies were raised on standard wheatmeal medium under a 12:12 h light:dark cycle and kept at 23 °C with 65 % humidity. Adult flies were collected upon eclosion with light CO2 anesthesia. Before mating, individual males or small groups of five to ten virgin females were kept in vials with fresh food. For laser vibrometry experiments, wings were removed so as to reduce noise produced by wings during grooming and fluttering in the recordings. Filming and laser vibrometry of courting pairs were performed at a temperature of around 23 °C.

### Recording Vibrational Signals with Laser Vibrometry

Video and laser vibrometer recordings were conducted on a vibration-damped table in a soundproof room. Flies were placed into cylindrical chambers of approximately 10 mm in diameter and 9 mm in height. The top of this cylinder was a transparent film through which the flies were recorded using a Stingray F-33B camera (Allied Vision). One side of the cylinder consisted of a piece of thermal foil, a membrane made of silver metallised polyester material, with an albedo of approximately 0.8 (Sub Zero Technology; Leicester, UK). The beam of a OFV-534 laser vibrometer (Polytec) was directed perpendicular to the surface of this membrane. Signals were digitised with 12bit amplitude resolution with a PCI MIO-16-E4 card (Analog Devices; Norwood, MA) and with LabView (National Instruments; Austin, TX) on a PC. Signals were transformed into .wav data with the Spike 2 (CED) or Neurolab (Hedwig and Knepper 1992) softwares. Video and laser vibrometer recordings were synchronised at the start by brief interruption of the laser path; this produces both a momentary peak in the oscillogram and a black frame in the video. Oscillograms were analysed with the Amadeus Pro (HairerSoft) and the Raven (Bioacoustics Research Program) softwares.

### Behavioral Recording of Courtship Assays

Pairs of flies were tested at 7 days old when they are most active in courtship. Their behavior was recorded with a 100 mm macro lens and a Stingray F-033B camera (Allied Vision Technologies; Stadtroda, Germany) and acquired with the Astro IIDC (Aupperle Services and Contracting; Calgary, Canada) or the Debut Video Capture (Pro Edition) softwares into a laptop computer. High-speed videos with images captured at a rate of 1000 per second were acquired with a Photron FastcamSA3 camera (Photron (Europe) Ltd., High Wycombe, Bucks, UK). Flies were filmed in transparent plexiglass courtship chambers (10 mm diameter and 9 mm height). Recording was started at the initiation of courtship and for approximately 600 s, or until copulation occurred. Each pair was tested only once. Before each test, chambers were washed with ethanol and dried.

### Behavior Annotations and Analysis

Movies were annotated with the Annotation software (Peter Brodsky, version 1.3), registering all standard male courting behaviors (that include orientating toward the female, following the female, moving and vibrating the wings, extending the proboscis, licking, leg tapping, etc.) and the exhibition of the postural display (that includes the wing-posture, movements and quivers of the abdomen, movements of the head and legs, production of liquid droplets, etc.), and also whether the female was moving or stationary. The data for each movie were imported into Excel files. We generated the box plots using the R program -BoxPlotR- from the Tyers Lab (http://boxplot.tyerslab.com/). The notches designate the 95 % confidence while the box limits specify the 25th and 75th percentiles. Bold middle lines indicate medians, and crosses indicate the means. Two-sample Wilcoxon tests were used to compare female movement (moving or stationary) during the postural display and to compare the interpulse intervals of the substrate-borne vibrations generated by *D. persimilis*, *D. pseudoobscura* and *D. melanogaster*.

### Behavioral Tracking during the Postural Display

Using Fabrice Cordelieres software, Manual Tracking, a plugin for imageJ (http://imagej.net/Manual_Tracking) we labeled the position of the abdomen, the mouth and the front-leg over time, resulting in the formation of three super imposed lines that trace the journey of these body parts over time as the movie plays forward. In the graph, zero represents the position of the body axis before the start of the postural display and each movement is relative to that original position.

### Sound Recordings

Sound was recorded using an insectavox composed of an electret microphone-amplifier board (frequency response, 50 Hz to 13 k Hz; sensitivity, 60 ± 3 dB; DC 3 V–12 V MAX9814) attached to an iMic USB audio device (griffintechnology.com, Nashville, USA) and a laptop computer. We used the software QuickTime (Apple inc.) for sound recording. Simultaneously, videotape of courtship was performed with a portable microscope USB camera (Visual Effect, model: B003). The audio files were processed using the audio editing program Audacity (http://audacityteam.org/).

## Results

### The male’s Courtship Parade and the female’s Response

#### The Male Display

**Fig. 1:**
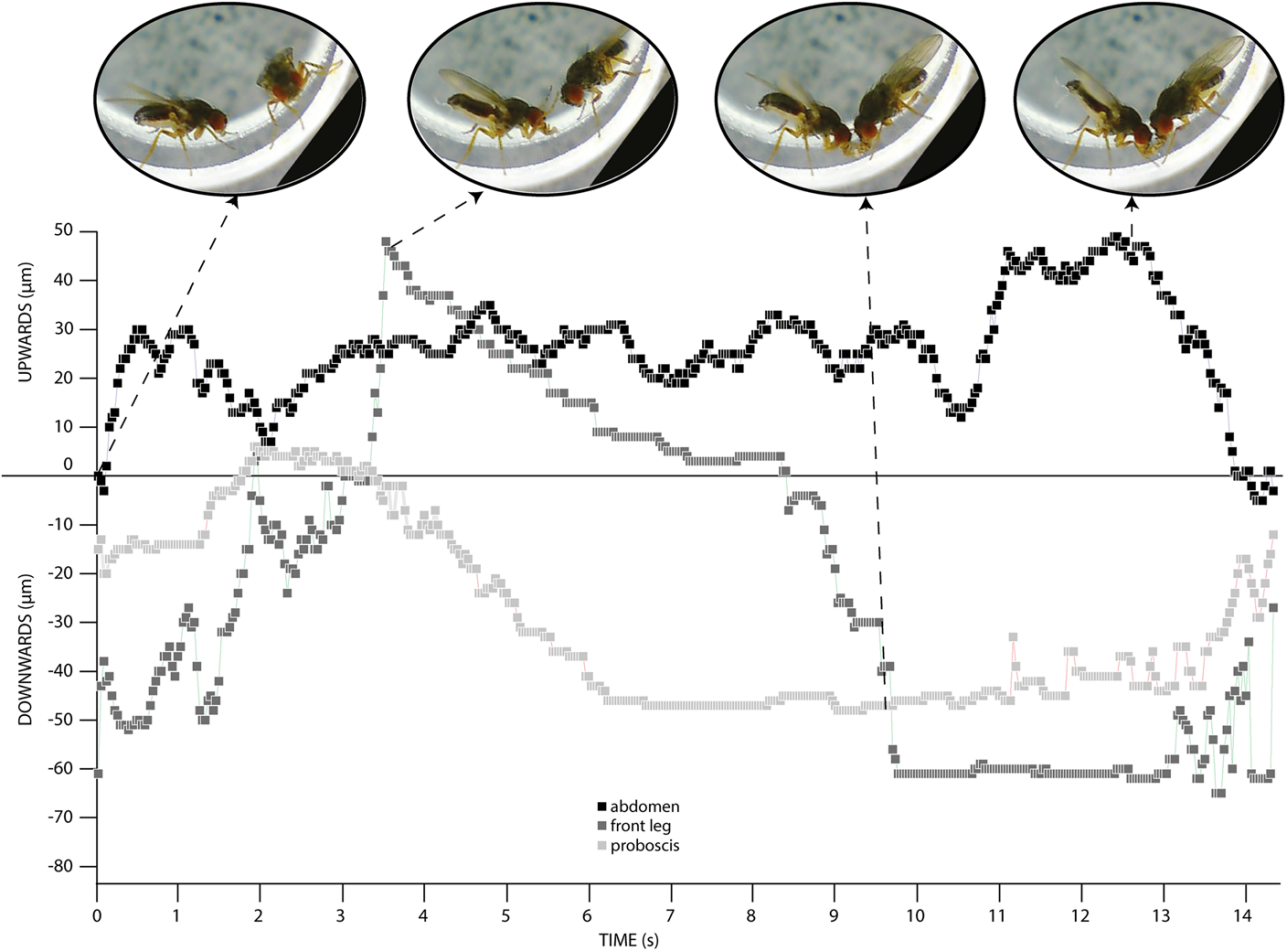
Visual tracking of three main behaviors within the PDC. We tracked the position of the tip of the abdomen (*in black*), one of the front legs (*in grey*) and the tip of the proboscis (*in light grey*) over time for a typical display bout (Supplementary Movie S1; 30 frames per second). Selected snapshots from the video clip are shown as landmarks. We used the position of the abdomen before the display as the position zero. The x axis shows time in seconds. The y axis shows distance in micrometres. For abdominal movements, the trace shows quivers within the larger abdominal upward and downward movements. During these behaviors the legs are raised and then lowered to the floor. The proboscis trace shows how the male moves his proboscis towards the ground where the female can touch the drop with her front legs and feed on it

We recorded 40 pairs of courting *D. persimilis* flies and monitored the postural display during courtship. We refer to this postural display of courtship as PDC. Using high-speed video imaging, we broke down the PDC into 12 behaviors (Supplementary Movie S1 and associated legend) and we tracked the movements of the male’s foreleg, abdomen and proboscis (Fig. 1, Supplementary Movie S2). The abdomen quivered while being progressively raised upward: we called this “quivering up” to differentiate it from the quivering that was observed during the standard courtship behaviors in other *Drosophila* species (Fig. 1; Supplementary Movies S1, S2) (Fabre et al. 2012). Sometimes, anal drops were produced (Supplementary Movie S3). The male tapped on the floor with its forelegs, and then raised one or both forelegs before moving it/them back towards the ground (Fig. 1, Supplementary Movies S1, S2, S3). While raising them up and down, the forelegs were also vibrating (Supplementary Movies S1, S2). Simultaneously, the male moved his proboscis downwards and produced a liquid droplet (Fig. 1, Supplementary Movies S1, S2, S3) presumably of regurgitated food with nutritional value (Steele 1986; Immonen et al. 2009), which was then collected by the female (Supplementary Movies S1, S3, S4).

#### The female’s Behavior during the male’s Display

**Fig. 2:**
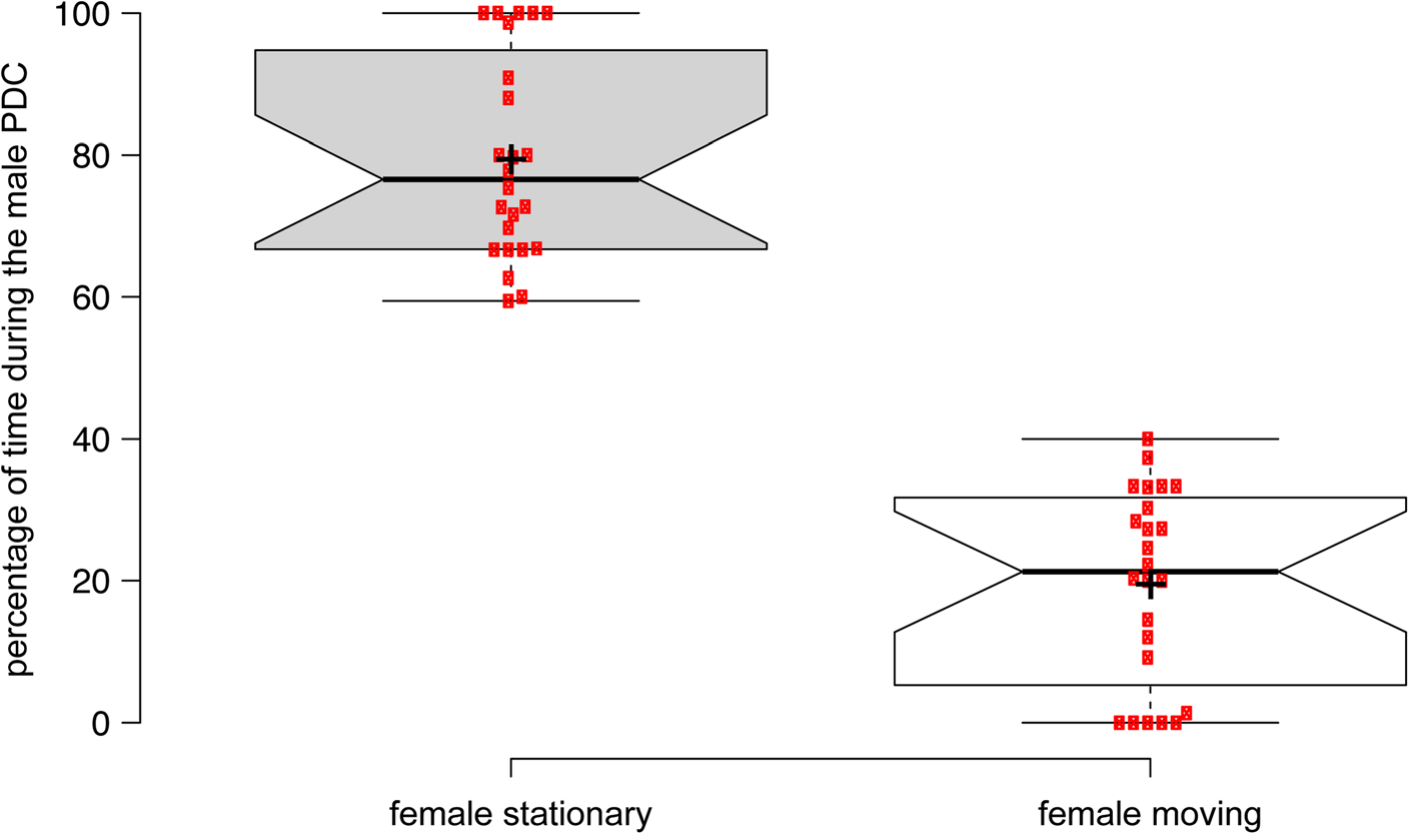
Quantification of the female movements during the PDC. Ethograms for males and females were constructed from video clips of 24 courting pairs. The box plots illustrate the percentage of time where females were moving (left box plot) or stationary (right box plot) while the male was performing the PDC. Females were more likely to be stationary when males performed the PDC (*n* = 24; *p* = 2.9 × 10–9). Thick lines indicate the medians and the notches indicate 95 % confidence intervals for each median; crosses indicate the mean values

During the male display, the female turned and faced the male (Supplementary Movies S1–4) but she remained mostly stationary (Fig. 2; during the PDC, the female was stationary for about 80 ± 3 % of the time; *n* = 24; *P* < 0.05). At the end of the display she approached the male to collect the droplet (Supplementary Movie S1–4). The female first touched the droplet with her forelegs (Supplementary Movies S1, S3), where flies have gustatory receptors (Montell 2009). Then, the labellar surface of her proboscis touched the labellar surface of the male’s proboscis in a “kiss” and she caught the liquid droplet directly (Supplementary Movies S1, S3). Alternatively, the female sometimes picked the droplet from the floor (Supplementary Movie S4). The female did not move while eating the droplet (Supplementary Movies S1, S4) and copulation could then occur (Supplementary Movie S4). If the female moved away or appeared not to attend to him, the male interrupted his behavior and copulation did not follow (Supplementary Movie S5).

#### Quantification of the PDC during *D. persimilis* Courtships

We found that in 47 % of the *D. persimilis* courting pairs (*n* = 40), the males exhibited both PDC and the standard *Drosophila* courtship behaviors, including fluttering one wing to produce a species-specific song, and extending the proboscis and tapping the female to assess her cuticular pheromones (see Movie S3; Spieth 1952). Within these pairs, courtship lasted an average of two minutes and the PDC occupied ~13 % of courtship time, predominantly occurring in the second half of courtship (not shown). Full PDC lasted between 10 and 20 s, with males exhibiting 2.6 ± 0.7 PDCs per courtship.

In the other 53 % of courting pairs, the males did not exhibit PDC and exhibited only the standard *Drosophila* courtship behaviors. In these courting pairs, the male did not produce regurgitated food. The “textbook view” is that *Drosophila* courtship is stereotyped (Greenspan and Ferveur 2000) and, to our knowledge, the finding that ~ half of the *D. persimilis* courting pairs behave differently to the other half has not previously been reported. We address this further in a separate report (manuscript *in preparation*).

### Substrate-Borne Vibrations Are Generated during the PDC

**Fig. 3:**
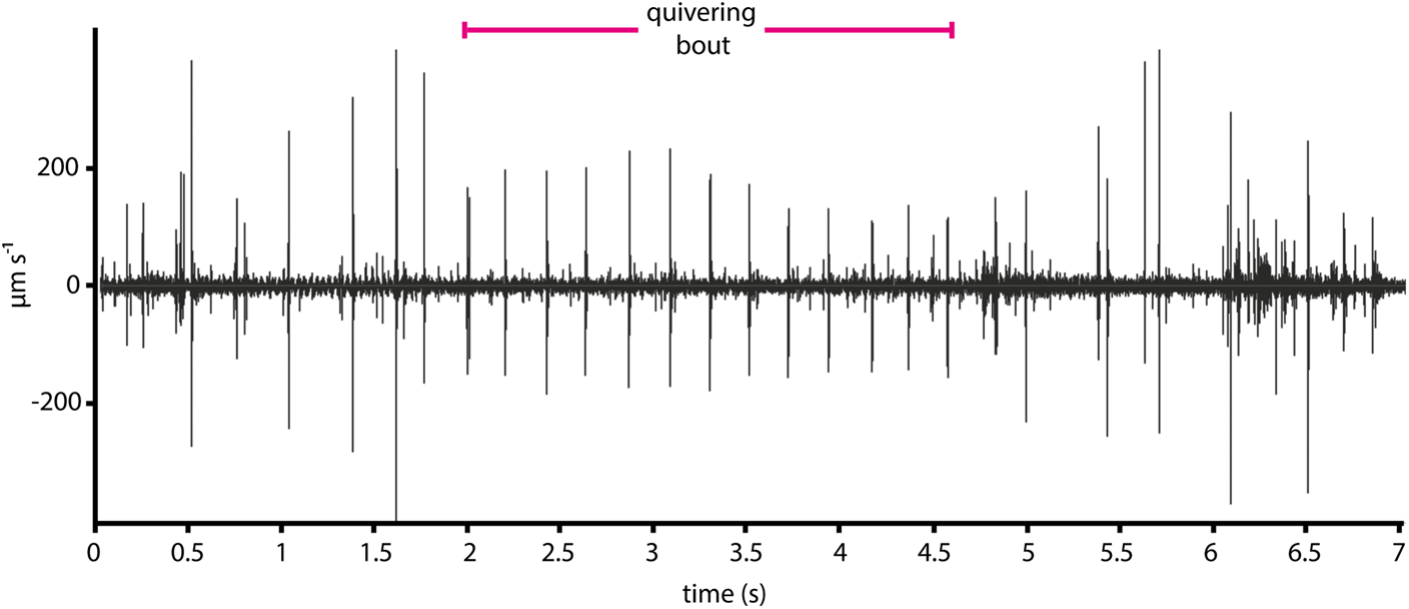
Regular substrate-borne vibrations are generated during the abdominal quivering-up of a courting *D. persimilis* male. Oscillogram of substrate-borne vibrations generated during a short single bout of quivering-up (Supplementary Movie S6 (at 42 s)); the wings of the male were amputated. Each pulse is about 5 ms long and of amplitude below 400 μm/s. Walking and grooming generate the larger amplitude signals visible before and after the quivering bout

**Fig. 4:**
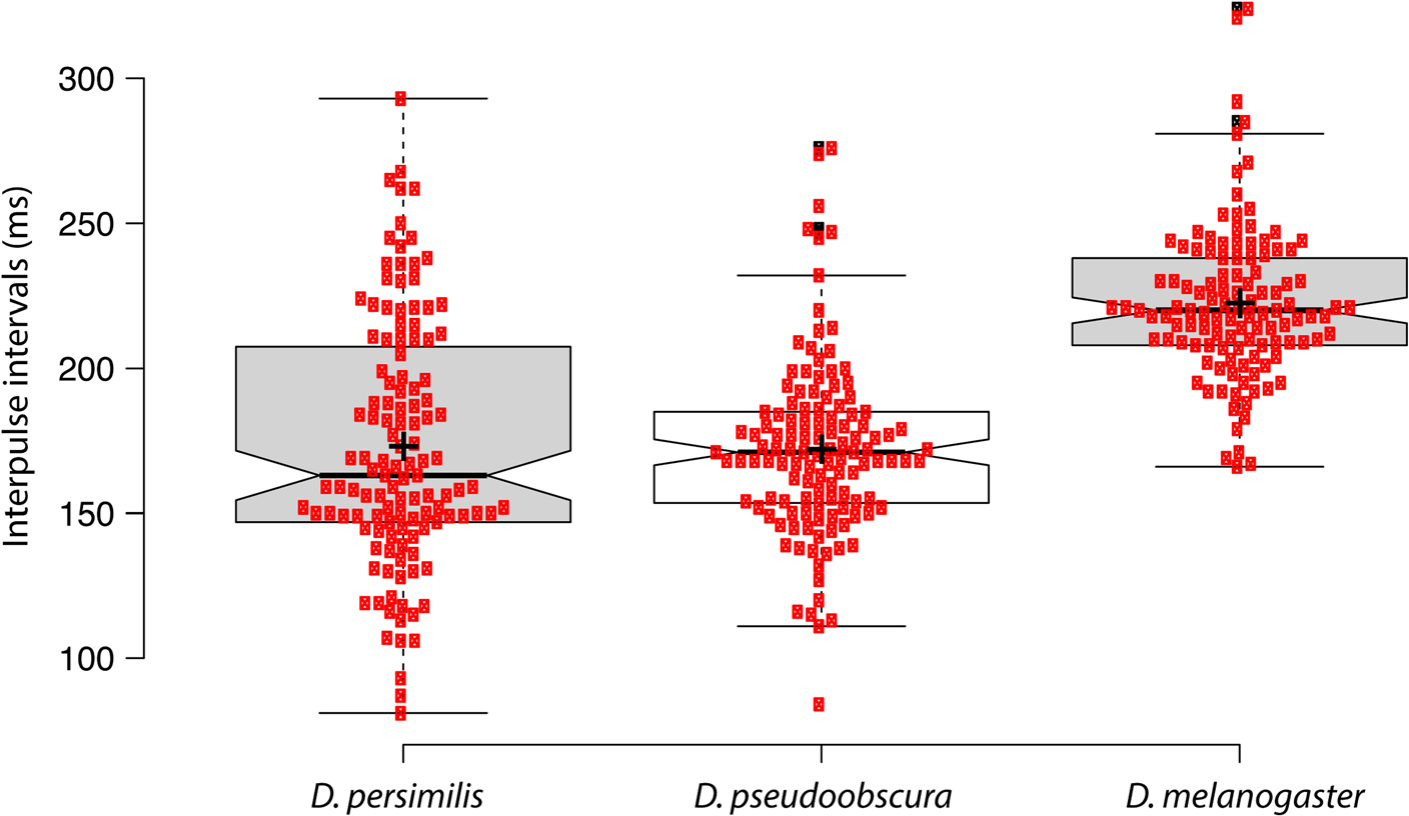
Interpulse intervals of the substrate-borne vibrations generated by quivering-up of *D. persimilis* and *D. pseudoobscura* males during the PDC and by quivering of *D. melanogaster* males during the standard courtship. Boxplots of the interpulse intervals (in ms) for the substrate-borne vibrations of wild-type *D. persimilis* males paired with wild-type *D. persimilis* virgin females (*n* = 124 pulses recorded, column 1); of wild type *D. pseudoobscura* males paired with wild type *D. pseudoobscura* virgin females (*n* = 125 pulses recorded; column 2); and wild type Canton S *D. melanogaster* males paired with Canton S virgin females (*n* = 117 pulses recorded; column 3). Data are shown for 3 individuals for each different species. There were no significant differences in the mean interpulse interval for *D. persimilis* and *D. pseudoobscura* species (*P* = 0.61). However, both showed significant differences with *D. melanogaster* mean interpulse interval *(P = 2.2 × 10–16). Red dots* show all data points. Notches indicate 95 % confidence intervals of each median, thick lines indicate the medians, and crosses indicate the mean values

Our aim was to identify which signals generated by the PDC may affect the female’s behavior. It appears that the display has a visual component because the male takes up a characteristic posture and the female turns to face him (Brown 1965) (Supplementary Movies S1–3). Nevertheless, we also investigated if quivering-up of *D. persimilis* generated vibrations in the substrate. We used a laser vibrometer to measure the oscillations in the substrate while observing the activity of the flies with a camera. We found that *D. persimilis* quivering-up generated regular substrate-borne vibrations (Fig. 3, Supplementary Movie S6) with an interpulse interval of 173 ± 4 ms (Figs. 3 and 4).

We compared the substrate-borne vibrations of *D. persimilis* males with those of *D. pseudoobscura,* a co-occuring (sympatric) species. *D. pseudoobscura* males also performed a PDC (Brown 1964), including abdominal quivering-up movements (not shown). Laser vibrometry recording revealed that these movements generated vibrations with similar interpulse intervals to *D. persimilis* (172 ± 3 ms; *P* = 0.61; Fig. 4). In contrast, however, these interpulse intervals were very different from those produced by *D. melanogaster* male quivering (222.4 ± 2 ms; Fig. 4; *P* < 0.05) (Fabre et al. 2012; Mazzoni et al. 2013; Medina et al. 2014).

### Air-Borne Sounds Do not Occur during the PDC

**Fig. 5:**
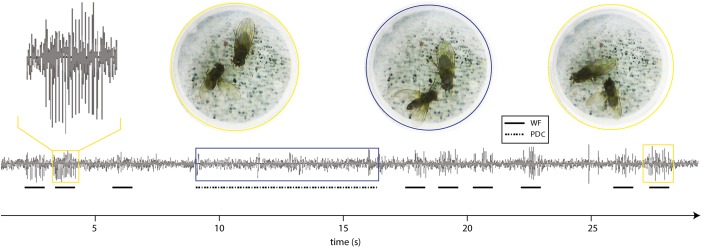
Insectavox sound recordings during *D. persimilis* courtship. Air-borne sounds were recorded during courtship using an insectavox (see materials and methods). Bouts of wing fluttering (WF) of *D. persimilis* males produced pulse song (*solid black lines*; supplementary Movie S7). Inset (*yellow box*) shows pulse song in more detail. The pulses have a frequency of around 200 Hz. After three bouts of WF, the male performed the PDC (indicated by a *blue rectangle* and a dotted line underneath). No characteristic air-borne signals were produced during the PDC (Supplementary Movie S7)

We examined if the wings produced air-borne cues during the wing-posture position; they move up-and-down during the display (Supplementary Movies S1–4). We did not detect any air-borne sound generated during the PDC, suggesting that these wing movements are silent (Fig. 5, Supplementary Movie S7). We did record, however, the pulse song generated by the male wing fluttering during the standard courtship behaviors (Waldron 1964; Noor and Aquadro 1998) (Fig. 5, Supplementary Movie S7).

## Discussion

Our results show that the postural display of courtship (PDC) produced by *D. persimilis* males is highly complex and includes twelve behaviors, some of which are performed simultaneously. In addition, the males use substrate-borne vibratory signals during the PDC, which co-occur with the upward and downward movement of the quivering abdomen. During these vibrations, the female remains stationary. The female is then proffered with a droplet that might be nutritive (Steele 1986; Immonen et al. 2009), which she feeds on while remaining immobile. Altogether, these results suggest that the male’s PDC endeavours to secure stationary females, as this may promote easier copulation (Ferveur 2010; Fabre et al. 2012). We do not know what is contained in the liquid droplet, although it is possible that it may promote egg laying (Steele 1986; Immonen et al. 2009). Females often touch this droplet with their front legs and this might allow them to assess its nutritive value or to evaluate male fitness or some other characteristic (Ferveur 2010; Tabadkani et al. 2012; Clutton-Brock and Huchard 2013). We were not able to find substrate-borne vibratory signals associated with the movement and vibration of the male’s forelegs. It is possible that the signals generated by the leg vibrations are concealed by the signals produced by the abdominal vibrations, as they are synchronous. Alternatively, the leg vibrations might only provide visual signals to the females. Similarly, during the parade of courting male jumping spider, *Habronattus dossenus,* substrate-borne vibratory signals were recorded during the abdominal vibratory movements, but not during some of the leg gestures (Elias et al. 2003).

The substrate-borne signals produced by *D. persimilis* males are different to those of *D. melanogaster*, indicating that the substrate-borne vibrations may be involved in species discrimination. However, the substrate-borne signals produced by *D. persimilis* males are similar to those of *D. pseudoobscura*. *D. persimilis* and *D. pseudoobscura* species do not produce hybrids in the wild (Dobzhansky and Epling 1944), even though they are morphologically similar, have similar cuticular hydrocarbons (Noor 1996), and produce similar substrate-borne signals (this study). Species boundaries are likely maintained by differences in the length of their genitalia (Rizki 1950) and the air-borne courtship songs that they produce (Brown 1965; Noor and Aquadro 1998); their evolutionary divergence has been much studied [see for example (Merrell 1954; Noor and Aquadro 1998; Williams et al. 2001; Noor et al. 2007)]. Our results suggest that the substrate-borne vibrations generated by *D. persimilis* and *D. pseudoobscura* males are unlikely to be involved in the discrimination between these two species and might instead constitute a common pacifying effect on the females in both species (Fabre et al. 2012).

It is likely that the female is attracted to the visual gestures of the PDC as she turns to face and observe the displaying male. Courtship in the dark would help reveal whether the substrate-borne vibratory signals are sufficient to account for the female’s attention during the PDC. Unfortunately, we obtained very low levels of *D. persimilis* and *D. pseudoobscura* courtship in the dark (not shown), something that was previously noted (Spieth 1952). This suggests that vision is important for their courtship, but it also makes it difficult to discriminate between the contribution of visual and substrate-borne signals for the female’s receptivity. Of note, however, is that the substrate-borne signals in some species of jumping spiders are more important than the visual displays for copulation success (Elias et al. 2005, 2010).

The abdominal quivering-up exhibited in *D. persimilis* during the PDC was reminiscent of the abdominal quivering reported in *D. melanogaster* and other *Drosophila* species during standard courtship behaviours (Fabre et al. 2012; Mazzoni et al. 2013) and these abdominal quivers all generate vibrations in the substrate. Unlike other species, however, *D. persimilis* males quiver while moving the abdomen upwards, away from the ground (Fabre et al. 2012; Mazzoni et al. 2013). When they were first identified it was unclear whether the substrate-borne vibrations generated by *D. melanogaster* males might depend on direct striking of the tip of the abdomen on the substrate (i.e. percussions) or whether they might be transmitted to the ground through the legs (i.e. tremulations) (Busnel et al. 1955; Morris 1980; Lasbleiz et al. 2006; Hill 2008; Fabre et al. 2012; Mazzoni et al. 2013). Clearly in *D. persimilis* abdominal striking on the substrate is not required to produce the substrate-borne vibrations. Close inspection of *D. melanogaster* abdominal quivers also suggests that the abdomen does not touch the substrate (Fabre et al. 2012; Mazzoni et al. 2013). Most likely therefore all abdominal quivers in *Drosophila* species are tremulations that are relayed to the substrate through the legs of the male or by other unknown mechanisms (Virant-Doberlet and Cokl 2004; Fabre et al. 2012; Mazzoni et al. 2013). The similar movements of tremulation (Busnel et al. 1955) through oscillation of body parts found in other animals for substrate-borne vibration production are thought to occur by coupling to the substratum through the adhesive hairs on the tips of the legs (Rovner 1980; Uetz and Stratton 1982; Aicher et al. 1983; Aicher and Tautz 1990; Dierkes and Barth 1995; Barth 2002; Elias et al. 2003; Uhl and Elias 2011).

Such a complex postural display that associates visual gestures and substrate-borne vibratory signals is unprecedented in *Drosophila*. It is reminiscent of the complex parades displayed by larger arthropods such as the jumping spiders (Elias et al. 2003; Hill 2008). It also adds to the growing list of examples showing that vibratory signalling is a mode of communication that is widespread among animals, in particular among arthropods, so much so that it has given rise to an entire field of study now known as “biotremology” (Endler 2014; Hill and Wessel 2016). It is unclear what triggers the PDC in *D. persimilis* males and why we observed it only in half of the courting pairs. Such an effortful display must presumably provide advantages to the male, but it is unknown how much more successful males producing PDCs are versus those that do not display. Further studies looking into different status of the courting pairs and different courting contexts should elucidate the favourable conditions in which *D. persimilis* males choose to exhibit the PDC during courtship, in addition to the standard courtship behaviors. Such studies will determine what benefits, if any, they gain from producing the PDC.

## Electronic supplementary material

ESM 1 Supplementary Movie S1 (MP4 24,661 kb)

ESM 2 Supplementary Movie S2 (AVI 5503 kb)

ESM 3 Supplementary Movie S3 (MP4 26,497 kb)

ESM 4 Supplementary Movie S4 (MP4 454 kb)

ESM 5 Supplementary Movie S5 (MP4 181 kb)

ESM 6 Supplementary Movie S6 (MP4 10,927 kb)

ESM 7 Supplementary Movie S7 (MP4 7200 kb)

ESM 8 (DOC 35 kb)
