# Supplementary material for: The Elaborate Postural Display of Courting Drosophila persimilis Flies Produces Substrate-Borne Vibratory Signals
Source: J Insect Behav. 2016 Sep 2;29(5):578–90. doi: 10.1007/s10905-016-9579-8 (PMC5055905; doi:10.1007/s10905-016-9579-8)
Supplement: Supplementary file 8 — (DOC 35 kb) [file 10905_2016_9579_MOESM8_ESM.doc]

**Examining the elaborate postural display of courting *Drosophila persimilis* flies reveals underlying seismic signals**

Mónica Vega Hernández^1^ and Caroline Cecile Gabrielle Fabre^1,*^

^1^Department of Zoology, University of Cambridge, Downing Street, Cambridge CB2 3EJ, United Kingdom

**Supplementary movie Legends**

**Supplementary Movie S1.** **A video clip of the PDC exhibited by *D. persimilis* males to females during courtship.**

The video is played first in real time, and then at 3 times slower.

12 behaviours characterise the display: The male stands by the female; the male extends his back legs and crouches on his front legs; he raises and then droops the abdomen; the male quivers the abdomen up-and-down; the male spreads his wings and takes up a “wing-posture”; the male moves his middle legs in rowing movements; the male raises one or both of his front legs up and down; the male moves his head downwards; the male extrudes his proboscis; the male produces a liquid droplet; the male feeds the female, directly (in some cases the feeding is made indirectly, see Supplementary Movie S4); in some cases the males produces an anal drop (see Supplementary Movie S3).

**Supplementary Movie S2.** **PDC** **with behavioural tracking, see Figure 1.**

This video clip shows the same clip as in Supplementary Movie S1 (played 3 times slower than normal speed) but now highlighting the movements of the abdomen (in blue), the front leg (in green), and the proboscis (in red) with a line and dot trace (see also Figure 1).

**Supplementary Movie S3.** **Several video clips showing *Drosophila* *persimilis* PDC and “standard” courtship behaviors.**

The first video clip shows the PDC viewed from underneath the flies. We see the liquid droplets produced by the male. The red arrow shows the anal droplet deposited onto the ground during the display. It is not clear what the function of this droplet might be; its deposition generates a brief substrate-borne signal (see Supplementary Movie S6 at 18s). The red circles indicate droplets produced by the proboscis.

The second video clip shows the PDC viewed from behind the male. The male curves his abdomen towards the ground and deposits the anal droplet (red arrow and indication). The male then displays a bout of “standard” *Drosophila* courtship parade (wing fluttering, proboscis extension, following the female, etc…) as is also displayed by *Drosophila melanogaster* males. Shortly after, the flies copulate.

The third video clip shows another example of PDC filmed with a high resolution/high speed camera (see materials and methods). The clip is played 30 times slower than the normal speed. Red arrows indicate the production of the anal droplet and its deposition on the ground. The female then touches the droplet that is produced through the male proboscis (red arrowhead) with her front leg (red arrowhead) before feeding on it. At the end of the movie a rowing movement of the middle leg can be seen.

**Supplementary Movie S4.** **A video clip showing variation in the feeding behaviour within the PDC.**

Sometimes the liquid droplet produced by the male is transferred directly to the female proboscis (see Supplementary Movies S1), but in other occasions the male deposits the droplet on the ground where the female retrieves it. Copulation often follows.

**Supplementary Movie S5.** **A video clip showing an aborted PDC.**

The interruption of the PDC coincides with the female turning and moving away from the male. The male does not produce the liquid droplet. Males will not complete their PDC if the female is not attentive.

**Supplementary Movie S6.** **A video and laser vibrometry recording of *D. persimilis* courtship showing that quivering-up coincides with regular substrate-borne vibrations (see Figure 3).**

A bout of courtship is shown. The flies are filmed from the side, and the laser vibrometer targets the substrate membrane on the top of the chamber, the membrane on which the flies stand. Oscillogram of the substrate-borne vibrations is played simultaneously at the bottom. Within this courtship bout, three PDC are displayed (at 3s, 10s, and 42s). The first PDC is incomplete. During the PDC, the male quivers-up and generates regular vibrations in the courting substrate that are recorded by the laser vibrometer (see also Figure 3). The third PDC has the most loud signal relative to noise because the male sits near to where the laser points. To reduce interference, the wings of the male were amputated, but the wing muscles still generate some substrate-borne vibrations of high frequencies (see Mazoni *et al.*, 2013). In this video, the sound you hear is a transformation of the oscillogram record to audible frequencies. The use of headphones rather than computer speakers is advised.

**Supplementary Movie S7. A video and insectavox (sound) recording of *D. persimilis* courtship showing when air-borne signals occur (see Figure 5).**

A bout of courtship is shown. The male extends one wing unilaterally. When the wing is vibrated up-and-down (flutters), it can generate a pulse song of around 200Hz, i.e. air-borne signals picked up by the insectavox microphone. When the male performs the PDC, including the quivering-up of its abdomen and the wing-posture (signalled by the appearance “quivering-up” in red), we could not detect any air-borne signal (except for some background noise that is made by the leg movements of the male and female on the microphone). The use of headphones rather than using the computer speakers is advised. See also Figure 5.
